# Identification of immune-related biomarkers associated with tumorigenesis and prognosis in cutaneous melanoma patients

**DOI:** 10.1186/s12935-020-01271-2

**Published:** 2020-05-25

**Authors:** Biao Huang, Wei Han, Zu-Feng Sheng, Guo-Liang Shen

**Affiliations:** 1 Department of Burn and Plastic Surgery, The First Affiliated Hospital of Soochow University, No. 188 Shizi Street, Suzhou, 215000 People’s Republic of China; 2 Department of Surgery, Soochow University, Suzhou, 215000 People’s Republic of China; 3 Department of Medicine, Soochow University, Suzhou, 215000 People’s Republic of China

**Keywords:** Cutaneous melanoma, Biomarker, Prognosis, Chemokines, Immune, Infiltration

## Abstract

**Background:**

Skin cutaneous melanoma (SKCM) is one of the most malignant and aggressive cancers, causing about 72% of deaths in skin carcinoma. Although extensive study has explored the mechanism of recurrence and metastasis, the tumorigenesis of cutaneous melanoma remains unclear. Exploring the tumorigenesis mechanism may help identify prognostic biomarkers that could serve to guide cancer therapy.

**Method:**

Integrative bioinformatics analyses, including GEO database, TCGA database, DAVID, STRING, Metascape, GEPIA, cBioPortal, TRRUST, TIMER, TISIDB and DGIdb, were performed to unveil the hub genes participating in tumor progression and cancer-associated immunology of SKCM. Furthermore, immunohistochemistry (IHC) staining was performed to validate differential expression levels of hub genes between SKCM tissue and normal tissues from the First Affiliated Hospital of Soochow University cohort.

**Results:**

A total of 308 differentially expressed genes (DEGs) and 12 hub genes were found significantly differentially expressed between SKCM and normal skin tissues. Functional annotation indicated that inflammatory response, immune response was closely associated with SKCM tumorigenesis. KEGG pathways in hub genes include IL-10 signaling and chemokine receptors bind chemokine signaling. Five chemokines members (CXCL9, CXCL10, CXCL13, CCL4, CCL5) were associated with better overall survival and pathological stages. IHC results suggested that significantly elevated CXCL9, CXCL10, CXCL13, CCL4 and CCL5 proteins expressed in the SKCM than in the normal tissues. Moreover, our findings suggested that IRF7, RELA, NFKB1, IRF3 and IRF1 are key transcription factors for CCL4, CCL5, CXCL10. In addition, the expressions of CXCL9, CXCL10, CXCL13, CCL4 and CCL5 were positively correlated with infiltration of six immune cells (B cell, CD8^+^T cells, CD4^+^T cells, macrophages, neutrophils, dendritic cells) and 28 types of TILs. Among them, high levels of B cells, CD8^+^T cells, neutrophils and dendritic cells were significantly related to longer SKCM survival time.

**Conclusion:**

In summary, this study mainly identified five chemokine members (CXCL9, CXCL10, CXCL13, CCL4, CCL5) associated with SKCM tumorigenesis, progression, prognosis and immune infiltrations, which might help us evaluate several immune-related targets for cutaneous melanoma therapy.

## Background

Skin cutaneous melanoma (SKCM) accounts for only 2% of total skin cancers. However, due to its high degree of malignancy and invasiveness, it causes over 72% of deaths in skin carcinoma [1]. The incidence of cutaneous malignant melanoma continues to increase annually [2]. Melanoma has become a serious public health problem, bringing great economic burden for society [3]. It is well known that melanoma is associated with multiple risk factors especially the sun exposure [4]. The general progression models of SKCM are from melanocyte to melanoma in situ, to invasive melanoma [5]. However, extensive research has explored the mechanism of recurrence and metastasis, the tumorigenesis of cutaneous melanoma remains unclear.

In the present study, we analyzed the differentially expressed genes (DEGs) between primary melanoma and normal skin to explore the potential tumorigenesis mechanism of SKCM. Our results mainly identified several chemokine family members (CXCL9, CXCL10, CXCL13, CCL4, CCL5) which were found related to better overall survival (OS) in SKCM patients. Chemokine family members are a group of low-molecular weight cytokines which were involved in many biological processes including angiogenesis, tumor development and metastasis, and the migration of leukocytes [6, 7]. In addition, we found that their expression levels were positively associated with infiltration of immune cells (CD4^+^T, CD8^+^T, B-cell, macrophages, neutrophils, dendritic cells) and tumor infiltrating lymphocytes (TILs). These immune infiltration cells play important roles in tumor microenvironment and can directly or indirectly regulate tumor immunity and modulate tumor immunological for anti-tumor effects [7, 8]. Therefore, our results may identify several immune-related biomarkers that may serve to guide SKCM therapy.

## Methods

### Patients and variables

A total of 46 melanoma and 46 normal tissues were obtained from 92 patients at the Department of Burn and Plastic Surgery, the First Affiliated Hospital of Soochow University (FAHSU, Suzhou, China) from March 2015 to August 2019. None of the patients had received radiotherapy or chemotherapy before operation. Tissue samples, including cutaneous melanoma and normal tissue, were collected during surgery and fixed in 4% paraformaldehyde, available from FAHSU tissue bank. Clinical data was available to obtain from hospital records. This research was supported by the Independent Ethics Committee (IEC) of the FAHSU and all patients were well informed of storing and upcoming use of their resected specimens for further research purposes.

### GEO and TCGA datasets

Expression profiling of SKCM patients with clinical information was obtained from the Gene Expression Omnibus (GEO) database (http://www.ncbi.nlm.nih.gov/geo) [9]. Among the inclusion criteria were (a) diagnosis of patients with primary melanoma (PM) and normal skin (NS), (b) detection of gene level in tissue or blood samples. Exclusion criteria included: (a) clinical data without survival time and outcome, and (b) datasets with small sample sizes (n < 15). Finally, three datasets were eligible: accession numbers GSE15605 (46 PM samples and 16 NS samples), GSE46517 (31 PM samples and 8 NS samples) and GSE114445 (16 PM samples and 6 NS samples). For validation, gene expression profiles of 472 melanoma patients were downloaded from the TCGA data portal (https://tcga-data.nci.nih.gov/tcga/) [10]. Clinical characteristics of patients were also obtained, including age, Clark level, Breslow depth, survival time, and outcome. All of the clinicopathological characteristics of SKCM patients were shown in Table 1.

**Table 1:** Clinicopathological characteristics of SKCM patients

Characteristics	FAHSU cohort (N = 46)	GEO cohort (N = 93)	TCGA cohort (N = 472)
N (%)			
Age			
≤ 60 years	21 (45.6)	53 (56.9)	258 (54.7)
> 60 years	25 (54.4)	40 (43.1)	214 (45.3)
Gender			
Male	30 (65.2)	55 (59.1)	297 (61.9)
Female	16 (34.8)	38 (40.9)	183 (38.1)
Clark level			
I	6 (13.0)	13 (13.9)	6 (1.8)
II	25 (54.3)	25 (26.8)	18 (5.5)
III–IV	12 (26.1)	47 (50.7)	246(75.5)
V	3 (6.6)	8 (8.6)	56 (17.2)
Breslow depth(mm)			
≤ 0.75	5 (10.9)	15 (16.1)	36 (10.2)
0.76–1.50	28 (60.9)	30 (32.2)	65 (18.4)
1.51–4.00	11 (23.9)	39 (41.9)	106 (30.0)
> 4.00	2 (4.3)	9 (9.8)	146 (41.4)
pT stage			
T1–T2	32 (69.6)	57 (61.3)	121 (32.7)
T3–T4	14 (30.4)	36 (38.7)	249 (67.3)
pN stage			
N0	46 (100)	93 (100)	236 (65.0)
N1	0 (0)	0 (0)	75 (20.7)
N2	0 (0)	0 (0)	52 (14.3)
pM stage			
M0	46 (100)	93 (100)	424 (94.4)
M1	0 (0)	0 (0)	25 (5.6)
Pathologic stage			
I–II	46 (100)	93 (100)	233 (53.9)
III–IV	0 (0)	0 (0)	199 (46.1)
Persistent distant metastasis			
No	46 (100)	93 (100)	217 (46.1)
Yes	0 (0)	0 (0)	254 (53.9)

SKCM, Skin Cutaneous Melanoma

FAHSU, The First Affiliated Hospital of Soochow University

GEO, The Gene Expression Omnibus

TCGA, The Cancer Genome Atlas

### Identification of DEGs

The DEGs between primary melanoma and normal skin were identified using GEO2R (http://www.ncbi.nlm.nih.gov/geo/geo2r) based on limma package with the threshold of|logFC| > 1 and P-value < 0.05. Next, the online Venn software (http://bioinformatics.psb.ugent.be/webtools/Venn/) was applied to detect the overlap DEGs among three datasets.

### DAVID database

To elucidate the biological functions of the overlapping DEGs, we performed functional annotation and pathway enrichment analysis via DAVID (The Database for Annotation, Visualization and Integrated Discovery, http://david.ncifcrf.gov/,version 6.8) online tool [11]. P-value < 0.05 was considered as the cutoff value. GO enrichment and KEGG pathway results were visualized as bubble charts by R software.

### STRING and cytoscape

STRING (http://string-db.org, version 11.0) database was used to predict the PPI network of DEGs and analyze the interactions between proteins [12]. An interaction with a combined score > 0.4 was recognized as statistical significance. The molecular interaction networks were visualized using the Cytoscape (version 3.7.0) and the most significant model in the PPI network was narrowed down by MCODE, with the following criteria: degree cutoff = 2, node score cutoff = 0.2, k-core = 2, max depth = 100 [13, 14].

### Functional enrichment

Metascape (https://metascape.org) was used to further verify the function enrichment of hub genes [15]. P < 0.05 was set as the cutoff value. Hub genes pathway analysis was performed and visualized by ClueGO (version 2.5.4) and CluePedia (version 1.5.4), the plug‐in of Cytoscape [16]. P-value < 0.01 was considered statistically significant.

### GEPIA database

GEPIA (http://gepia.cancer-pku.cn/index.html) is an analysis tool containing RNA sequence expression data of 9736 tumors and 8587 normal tissue samples [17]. In this study, GEPIA was used to perform survival analysis based on the data from TCGA database. Student’s t test was used to analyze the correlation between the expression and pathological stages. P value < 0.05 was considered statistically significant.

### Immunohistochemistry (IHC)

Protein expression levels of hub genes were measured using IHC staining and rabbit polyclonal anti-CCL4 antibody (ab235978), anti-CCL5 antibody (ab9679), anti-CXCL9 antibody (ab9720), anti-CXCL10 antibody (ab9807), anti-CXCL13 antibody (ab272874). Positive or negative staining of a certain protein in one FFPE slide was independently assessed by two experienced pathologists and supervised by a clinician. Based on the staining intensity level (no staining, weak, moderate and strong staining), the score was ranging from 0 to 3, as previous described. The staining extent was graded from 0 to 4 for the coverage percentage of immunoreactive tumor cells (0%, 1–25%, 26–50%, 51–75%, 76–100%). The overall IHC score grading from 0 to 12 was evaluated according to the multiply of the staining intensity and extent score. Negative staining represented grade 0 to 4 and positive staining from 5 to 12 for each sample.

### cBioPortal database

The cBioPortal database (www.cbioportal.org), a comprehensive web resource, can visualize and analyze multidimensional cancer genomics data [18]. The genetic alterations of prognostic genes were obtained from cBioPortal based on 475 SKCM samples in TCGA.

### TRRUST database

TRRUST (https://www.grnpedia.org/trrust/) is a useful predict tool for human, and mouse transcriptional regulatory networks [19]. The TRRUST database can provide information on how these interactions are regulated, containing 8444 transcription factor (TF)-target regulatory relationships of 800 human TFs.

### Immune infiltration analysis

Prognostic gene expression data was used to measure the abundance of six types of infiltrating immune cells (B cells, CD4^+^T cells, CD8^+^T cells, neutrophils, macrophages, and dendritic cells) in SKCM patients using the Tumor Immune Estimation Resource (TIMER) algorithm [20]. Then, an integrated repository portal for tumor-immune system interactions (TISIDB, http://cis.hku.hk/TISIDB/index.php) was utilized to examine tumor and immune system interactions in 28 types of TILs across human cancers [21]. Spearman’s test was used to measure correlations between prognostic genes and TILs. All hypothetical tests were two-sided and P-values < 0.05 were considered statistically significant.

### The drug–gene interaction database

DGIdb (version 2.0) is an open-source project that help users mine existing resources and generate assumptions about how genes are therapeutically targeted or prioritized for drug development [22]. The parameters were set as: preset filters: FDA approved; antineoplastic; all the default.

### Statistical analysis

The expression heatmap and correlation coefficient were analyzed and visualized by pheatmap and corplot packages in R software. ROC curve of prognostic genes was performed by SPSS 22.0. P-values < 0.05 were considered statistically significant in all tests.

## Results

### DEGs selection

**Fig. 1:**
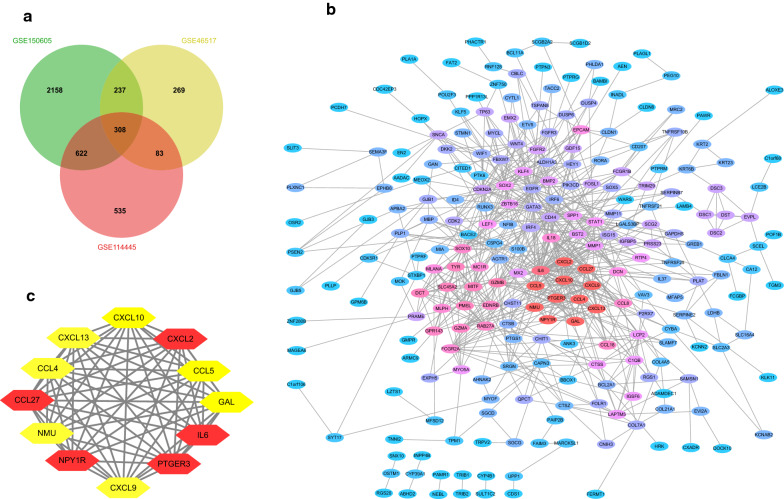
Venn diagram, PPI network and the most significant module of DEGs. **a** The three datasets explored an overlap of 308 genes in Venn diagram. **b** The PPI network of DEGs was performed by Cytoscape. **c** The most significant module of DEGs was obtained from PPI network with 12 nodes. (DEGs, differentially expressed genes; PPI, protein‐protein interaction)

After standardization and identification of the microarray results, DEGs (4640 in GSE15065,1096 in GSE46517 and 1895 in GSE114445) were selected. The overlap among three datasets included 308 genes as shown in the Venn diagram (Fig. 1a, Additional file 1: Table S1) between primary melanoma and normal skin.

### GO and KEGG enrichment analysis of the DEGs

**Fig. 2:**
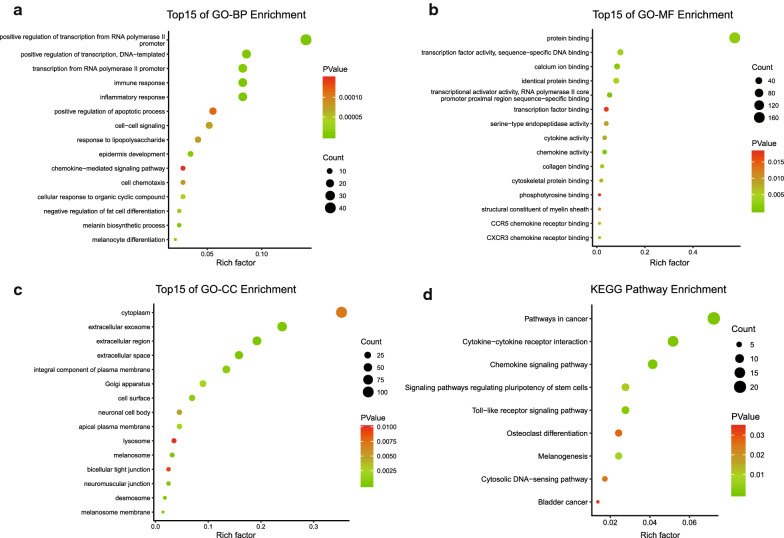
GO and KEGG analysis of the overlapping DEGs in SKCM. **a** Biological process. **b** Cellular component. **c** Molecular function. **d** KEGG pathway. Functional and pathway enrichment of DEGs was performed by DAVID, displayed in bubble charts

Functional and pathway enrichment of DEGs was performed by DAVID, then displayed in bubble charts by R software (P < 0.05, Fig. 2). GO analysis indicated that changes in biologic processes were significantly enriched in the inflammatory response, immune response, regulation of transcription. As for cellular component, DEGs were particularly enriched in extracellular region, extracellular space and extracellular exosome. Changes in molecular function were mostly enriched in chemokine activity, transcriptional activator activity, protein binding and CXCR3 chemokine receptor binding. KEGG pathway analysis demonstrated that DEGs played pivotal roles in pathways in cytokine–cytokine receptor interaction, chemokine signaling pathway and pathways in cancer. These results revealed that immune response and inflammatory response play important roles in SKCM tumorigenesis.

### PPI network construction and hub genes identification

The PPI network was constructed via Cytoscape, including 247 nodes and 792 edges (Fig. 1b). Then, the most important PPI network module was obtained using MCODE, consisted of 12 nodes and 64 edges (Fig. 1c). After selection, CCL5, PTGER3, IL6, CXCL13, CCL27, CCL4, CXCL9, NMU, CXCL2, GAL, NPY1R, CXCL10 were considered as hub genes of the network.

### Function analysis of hub genes

**Fig. 3:**
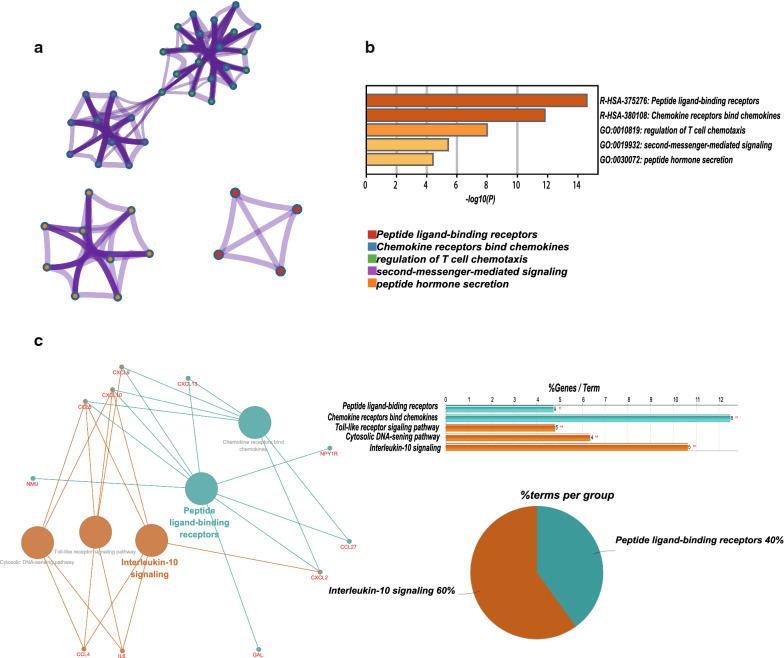
The function analysis of hub genes (P < 0.01). **a**, **b** The functions of hub genes were mainly enriched in regulation of T cell chemotaxis, peptide ligand-binding receptors and chemokine receptors bind chemokine signaling. **c** The most significant pathway and related genes

As expected, functional annotation obtained from Metascape suggested that hub genes were mainly enriched in regulation of T cell chemotaxis, peptide ligand-binding receptors and chemokine receptors bind chemokine signaling (Fig. 3a, b, P < 0.001). Similarly, ClueGO (Fig. 3c, P < 0.01) revealed that the most involved pathways were interleukin-10 signaling, peptide ligand-binding receptors and chemokine receptors bind chemokine signaling.

### The prognostic and diagnostic value of hub genes in SKCM patients

**Fig. 4:**
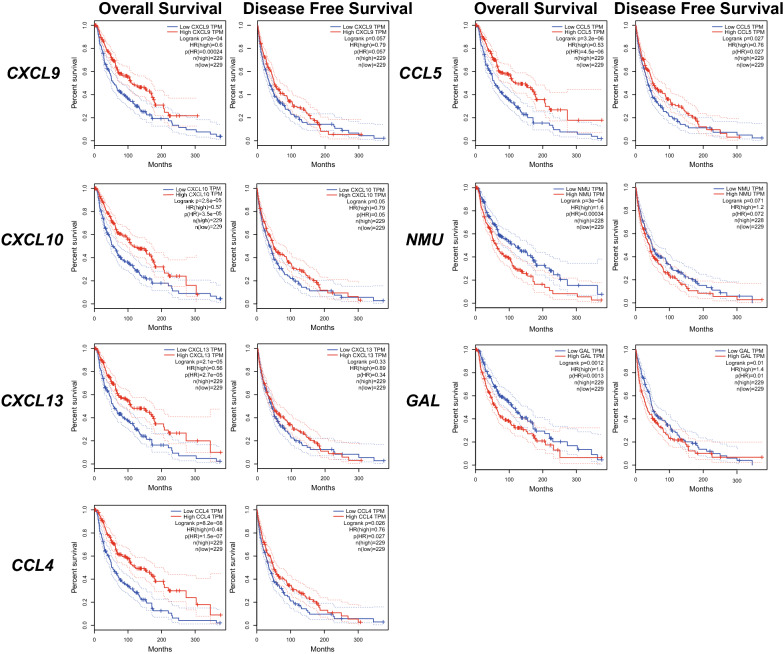
The prognostic value of five chemokines (CXCL9, CXCL10, CXCL13, CCL4, CCL5) and NMU, GAL in SKCM patients in the overall survival and disease-free survival curve

**Fig. 5:**
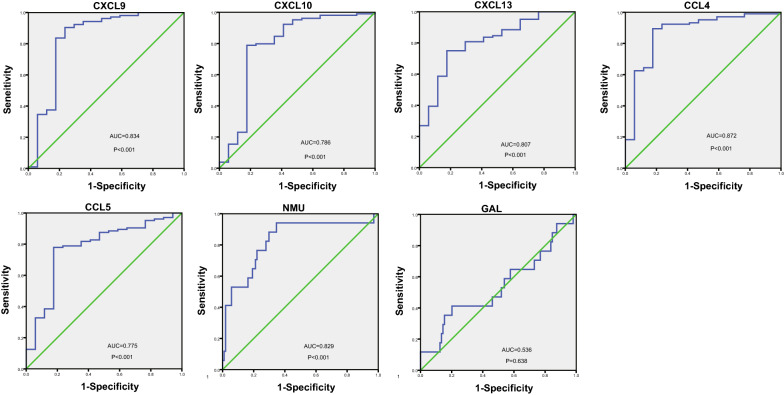
Assessment of diagnostic value by ROC curves. Results showed CCL4 (AUC = 0.872), CCL5 (AUC = 0.775), CXCL9 (AUC = 0.834), CXCL10 (AUC = 0.786), CXCL13 (AUC = 0.807), NMU (AUC = 0.829) except GAL (AUC = 0.536) were of diagnostic value with p value < 0.001

Using the Kaplan‑Meier method, the prognostic values of the hub genes in SKCM patients were determined. Seven hub genes were significantly associated with OS as shown in Fig. 4. High expression of CXCL9 (HR = 0.6; P = 2E−4), CXCL10 (HR = 0.57; P = 2.8E−5), CXCL13 (HR = 0.56; P = 2.1E−5), CCL4 (HR = 0.48; P = 8.2E−8), CCL5 (HR = 0.53; P = 3.2E−6) were significantly associated with better OS. In contrast, NMU (HR = 1.6; P = 3E−4) and GAL (HR = 1.6; P = 0.001) were found associated with poor overall survival. Furthermore, CCL4 (HR = 0.76; P = 0.026), CCL5 (HR = 0.76; P = 0.027) were also found significantly associated with better disease-free survival (Fig. 4). Except GAL (AUC = 0.536), the rest of hub genes, including CCL4 (AUC = 0.872), CCL5 (AUC = 0.775), CXCL9 (AUC = 0.834), CXCL10 (AUC = 0.786), CXCL13 (AUC = 0.807) and NMU (AUC = 0.829), were of diagnostic value (P < 0.001) (Fig. 5).

### Validation the aberrant expression of prognostic genes and their clinical characteristics

**Fig. 6:**
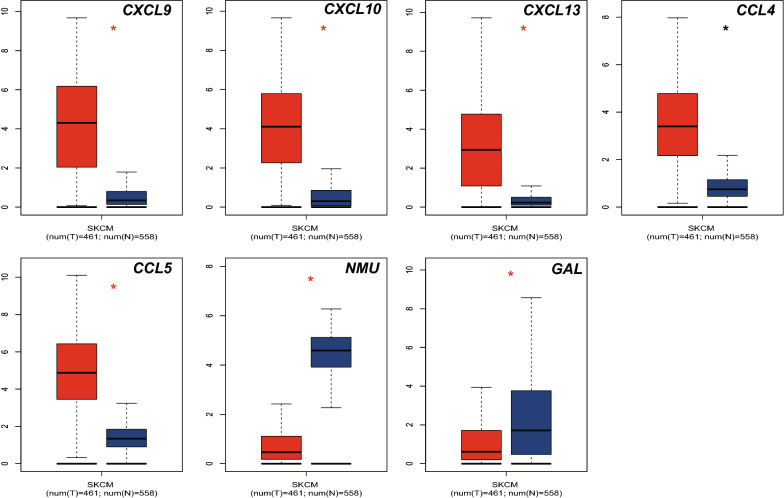
The transcriptional levels of CXCL9, CXCL10, CXCL13, CCL4, CCL5, NMU and GAL were significantly difference in SKCM vs normal skin tissues. The P value was set at 0.05

Based on data from GEPIA database, all of the prognostic genes were found differential expressed in SKCM vs normal skin tissue (P < 0.05, Fig. 6).

**Fig. 7:**
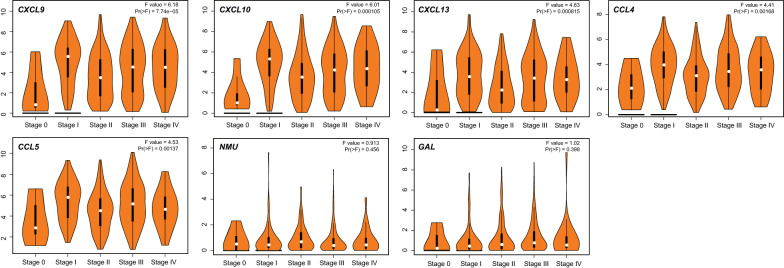
CXCL9, 10, 13 and CCL4, 5 were significantly overexpressed in stage I and decreased in subsequent stages (P value < 0.001)

In addition, CXCL9 (P = 7.74E−5), CXCL10 (P = 1.05E−4), CXCL13 (P = 8.15E−4), CCL4 (P = 0.002), CCL5 (P = 0.001) were significantly correlated to SKCM pathological stages (Fig. 7). Among them, CXCL9, CXCL10, CXCL13, CCL4, CCL5 were higher expressed in stage I and decreased in the subsequent stages.

**Fig. 8:**
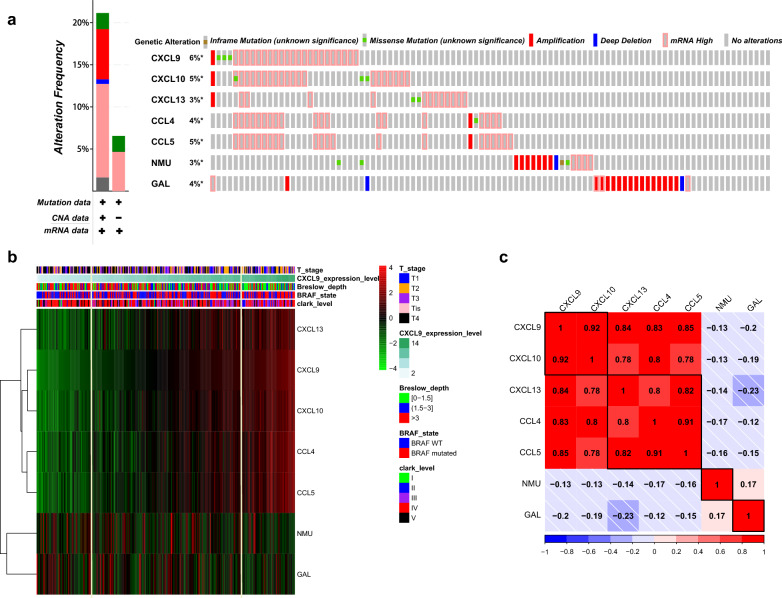
Genetic alteration, neighbor gene network, and interaction analyses of prognostic genes in SKCM patients. **a** Summary of alterations in prognostic genes in SKCM. **b** The expression heatmap and the relationship of clinical outcomes among prognostic genes. **c** Correlation heat map of prognostic genes

Subsequently, all of the samples from TCGA were ordered according to the expression level of CXCL9, and statistical analysis was conducted to compare the head 80 with the tail 80 samples. The heatmap (Fig. 8b) and statistical results suggested that higher expression levels (tail 80 samples) of CXCL9, CXCL10, CXCL13, CCL4, CCL5 were related to more patients of lower Breslow depth (0–1.5 mm, 51.2%), BRAF mutation (60%) and lower Clark levels (I–III 45% and IV–V, 55%), reduced T4 stage (T4, 25%). With the decrease levels of these five chemokines (head 80 samples), there were more patients with higher Breslow depth (0–1.5 mm, 15% and > 1.5 mm, 85%), BRAF WT (wild type, 56.3%), higher Clark levels (I–III 17.5% and IV–V, 82.5%) and T4 stage (T4, 47.5%).

All of the data above suggested that five chemokine family members play critical roles in cutaneous melanoma tumorigenesis and progression.

### Genetic alteration, correlation coefficient and key transcription factors analyses of prognostic genes in patients with SKCM

As a result, there were nearly 6% (CXCL9), 5% (CXCL10), 3% (CXCL13), 4% (CCL4), 5% (CCL5), 3% (NMU), 4% (GAL) of SKCM samples had genetic alteration. The most common genetic change among five chemokines (CXCL9, CXCL10, CXCL13, CCL4, CCL5) was enhanced mRNA expression (Fig. 8a). While genetic change in GAL and NMU were mainly related to amplification.

Then, we assessed the correlation coefficients in prognostic genes. As expect, significant positive correlations were observed between five chemokines (Fig. 8c). Among them, CXCL9 and CXCL10 had the highest positive correlation with a Spearman’s correlation coefficient of 0.92. CXCL9 exhibited the tightest association with all other chemokines, with a median Spearman’s correlation coefficient of 0.85.

Using TRRUST database, we explored potential transcription factor targets of the five chemokines. As a result, IRF7, IRF3 (FDR = 3.09E−05); RELA, NFKB1 and IRF1 (FDR = 1.5E−04) were found to be the key transcription factors for CCL4, CCL5, CXCL10 (Table 2).

**Table 2:** Key transcriptional factors (TFs) of five chemokines in SKCM (TRRUST database)

Key TF	Description	P-value	FDR	Regulated genes
IRF7	Interferon regulatory factor 7	7.77E−06	3.09E−05	CCL5, CXCL10
IRF3	Interferon regulatory factor 3	1.24E−05	3.09E−05	CXCL10, CCL5
RELA	v-Rel reticuloendotheliosis viral oncogene homolog A (avian)	0.000134	0.000149	CCL4, CXCL10, CCL5
NFKB1	Nuclear factor of kappa light polypeptide gene enhancer in B-cells 1	0.000137	0.000149	CCL5, CXCL10, CCL4
IRF1	Interferon regulatory factor 1	0.000149	0.000149	CXCL10, CCL5

### Validation of five chemokines expression in SKCM tissues from the FAHSU cohort

**Fig. 9:**
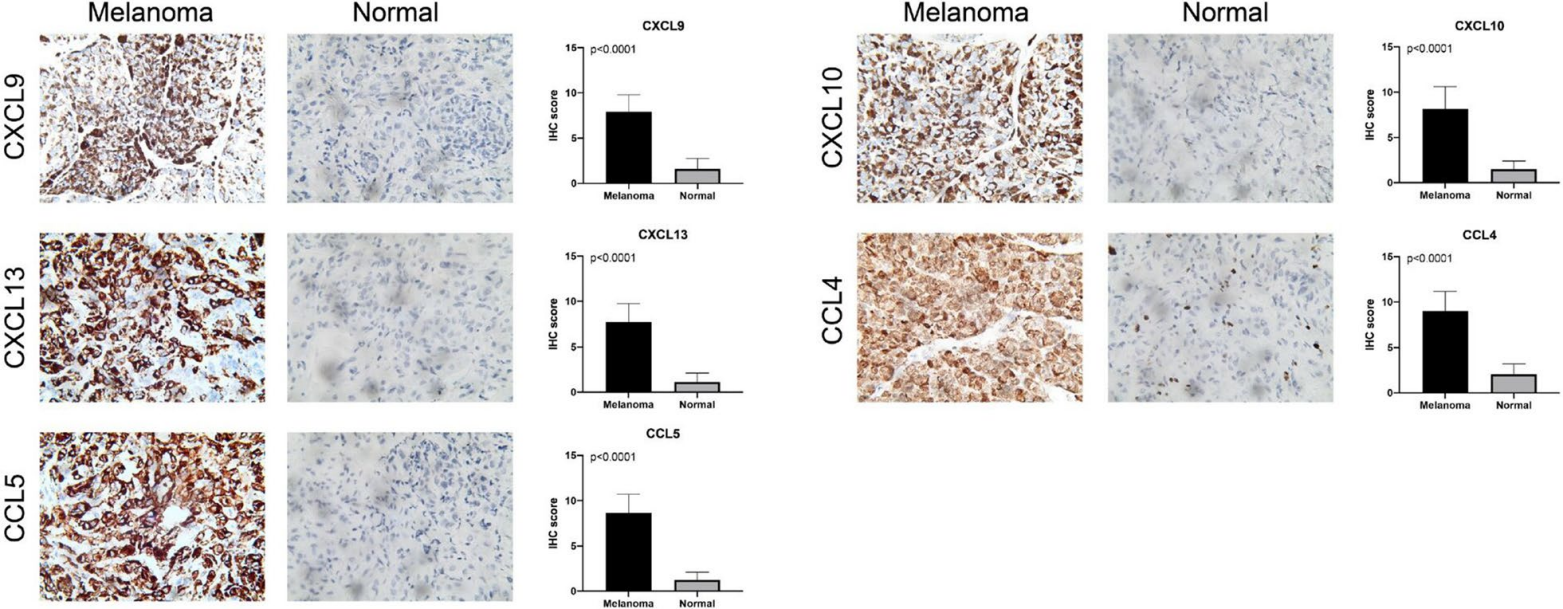
Validation of five chemokines expression in SKCM tissues from the FAHSU cohort. IHC staining indicated significantly elevated expression of CXCL9, CXCL10, CXCL13, CCL4, CCL5 in SKCM tissues compared with normal tissues (P < 0.0001)

Therefore, IHC was performed to validate the expression of five chemokine family members. Consistent with the mRNA expression, we found significantly elevated CXCL9, CXCL10, CXCL13, CCL4 and CCL5 proteins expression in the SKCM than in the normal tissues. The results and the plots of IHC score were illustrated in Fig. 9.

### Immune infiltration analysis

**Fig. 10:**
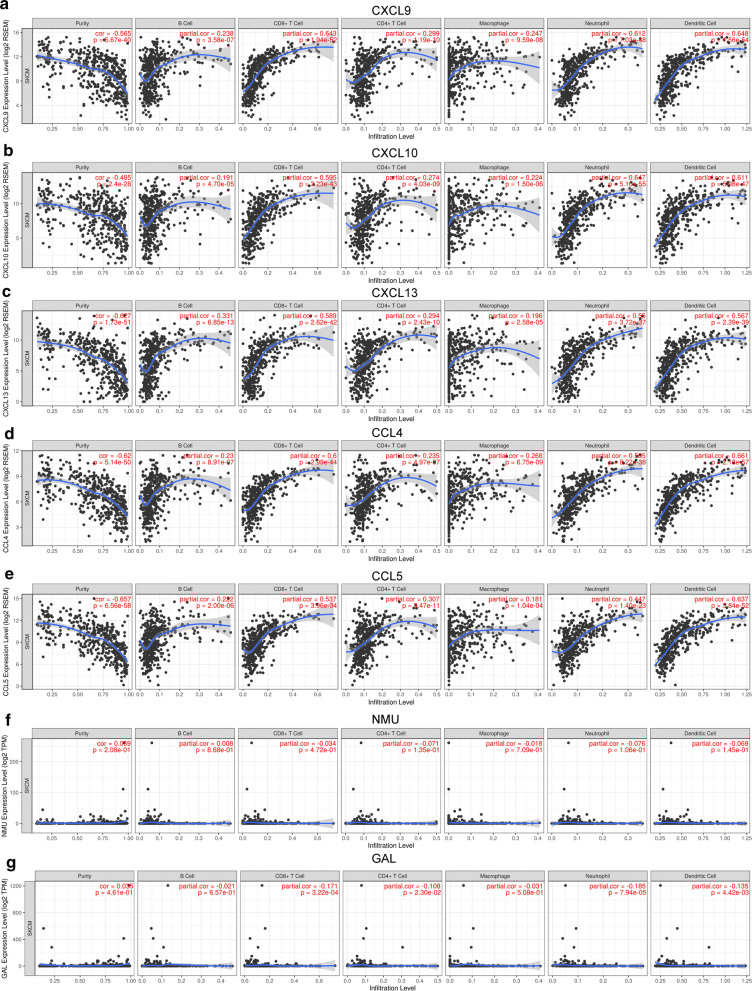
The correlation between prognostic genes expression and immune cell infiltration (TIMER). The correlation between the abundance of immune cell and the expression of **a** CXCL9; **b** CXCL10; **c** CXCL13; **d** CCL4; **e** CCL5; **f** NMU; **g** GAL

We performed correlation analysis between prognostic genes expression and immune infiltration level for SKCM. There was a positive correlation between CXCL9 expression and the infiltration of B cells (Cor = 0.238, p = 3.58e−07), CD8+ T cells (Cor = 0.643, p = 1.94e−52), CD4+ T cells (Cor = 0.299, p = 1.19e−10), macrophages (Cor = 0.247, p = 9.59e−8), neutrophils (Cor = 0.612, p = 7.03e−48), and dendritic cells (Cor = 0.648, p = 1.56e−54; Fig. 10a). Similar results were obtained for CXCL10, CXCL13, CCL4 and CCL5 (Fig. 10b–e). While, the correlation between NMU/GAL and immune infiltration is not obvious (Fig. 10f, g). Additionally, we also found significant correlations of these seven genes with 28 types of TILs across human heterogeneous cancers (Additional file 2: Fig. S1). CXCL9 significantly positive correlated with abundance of 28 types TILs such as activated CD8 T cells (Act_CD8 T cells; rho = 0.801, P < 2.2e−16), regulatory T cells (Treg; rho = 0.693, P < 2.2e−16), macrophage (rho = 0.655, P < 2.2e−16), natural killer T cells (NK T cells; rho = 0.74, P < 2.2e−16), myeloid derived suppressor cells (MDSC; rho = 0.744, P < 2.2e−16), immature B cell (Imm_B; rho = 0.78, P < 2.2e−16). Similar results were found in CXCL10, CXCL13, CCL4 and CCL5 (Additional file 2: Fig. S1).

Additionally, in the survival model of TIMER, results showed that high levels of B cells (Log-rank P = 0), CD8+ T cells (Log-rank P = 0), neutrophils (Log-rank P = 0), dendritic cells (Log-rank P = 0), CXCL9 (Log-rank P = 0), CXCL10 (Log-rank P = 0), CXCL13 (Log-rank P = 0), CCL4 (Log-rank P = 0), CCL5 (Log-rank P = 0), were significantly related to longer survival time in SKCM patients (Additional file 3: Fig. S2).

### Drug–gene interaction prediction

**Fig. 11:**
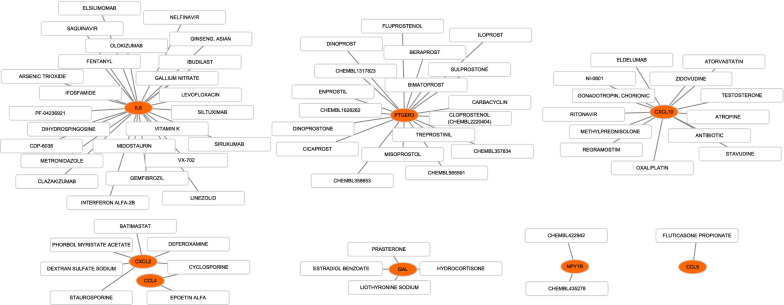
Drug–gene interaction diagram, yellow circle indicates the related hub gene and white square indicates the drug

We obtained 42 drug-gene interaction pairs in DGIdb, including genes (IL6, CXCL2, CXCL10, CCL4, CCL5, PTGER3, GAL, NPY1R) and 69 drugs, as shown in Fig. 11. This result may help develop new targets for SKCM therapy.

## Discussion

Skin is the largest organ of human beings. There are about 1500 melanocytes in human epidermis per square millimeter, equivalent to nearly 3 billion melanocytes in an ordinary human skin [23]. The incidence of SKCM continues to increase every year. Once melanoma spreads through the dermis, the prognosis is poor and melanoma is not projected to reduce the death rate in the next few years [24]. A series of therapeutic methods, such as radiotherapies, chemotherapies, targeted therapies and immunotherapies have enhanced advanced patient survival [25]. However, many patients that are being administered these therapies demonstrate a low durable response, drug resistance and poor prognosis [26]. Therefore, more therapeutic targets and prognostic biomarkers must be identified.

In our study, 308 DEGs were identified between primary SKCM and normal skin, and the functional annotation indicated that inflammatory response and immune response were closely associated with SKCM tumorigenesis. KEGG pathways in hub genes included IL-10 signaling, chemokine receptors bind chemokine signaling and peptide ligand-binding receptors. STAT3 is essential for the action of IL-10 and IL-6. IL-6 plays a central role in melanoma development and enhances the IL-10 production via STAT3-dependent signaling [27]. IL-6,10/JAK-STAT3 pathways are found in many carcinomas, and their hyperactivation was generally related to unfavorable clinical prognosis [28]. IL-6 and IL-10 create a favorable environment to support tumorigenesis by increasing the cancer cell proliferation, angiogenesis, metastasis and contribute to enhancing the immune suppression [28]. Smith et al. found that IL-10 directly inhibits CD8^+^T cell function by enhancing N-Glycan branching to decrease antigen sensitivity [29]. However, chemokine and its receptors usually attract immune cells such as CD8^+^T cell and NKs in the tumor microenvironment for immune activation [30]. Apparently, these two pathways play opposite roles in tumor formation, and the tumorigenesis of SKCM may be related to their abnormality.

Moreover, SKCM patients with high expression of five chemokines (CXCL9, CXCL10, CXCL13, CCL4, CCL5) were associated with better OS. While high levels of NMU and GAL showed poor prognosis. In addition, we found that the high expression of CXCL9, CXCL10, CXCL13, CCL4 and CCL5 were related to better outcomes in Breslow depth, Clark level, T-stages. IHC results from FAHSU cohort verified that CXCL9, CXCL10, CXCL13, CCL4, CCL5 were significantly over-expressed in SKCM tissues. All of the results suggested that chemokines members (CXCL9, CXCL10, CXCL13, CCL4, CCL5) and NMU, GAL were important biomarkers in SKCM tumorigenesis, progression and prognosis.

Chemokines are chemotactic cytokines mediating the migration and localization of immune cells [31]. C–X–C motif ligand 9,10,13 (CXCL9, CXCL10, CXCL13) belong to CXC family chemokine. They are predominantly expressed by immune cells such as macrophages and T cells. CXCL9, CXCL10 exert their function through binding to C-X-C motif receptor CXCR3 that was highly expressed on the activated T cells [30]. Research shows that CD8^+^T cell infiltration is CXCR3 dependent [32]. CD8^+^T cell plays important role in tumor microenvironment, which inhibits the proliferation and metastasis of tumor cells. A recent research found that the CXCL10-CXCR3 axis may relate to melanoma brain metastasis [33]. Additionally, the CXCR3 ligands enhanced the response rates to immune checkpoint blockade (anti-PD-1, anti-CTLA-4) by recruiting T cells [34]. These results reveal the importance of manipulating this axis, which provide us opportunities to manipulate chemokine expression for tumor therapy and prevention.

C–C motif ligand CCL4, CCL5 belong to CC family chemokine. CCL4, CCL5, CXCL9, CXCL10 were confirmed to be preferentially expressed in tumors that contained T cells [35]. This suggests that CCL4, 5 and CXCL9,10 may act in the same pathway in T-cell recruitment. The experimental results carried out by Michelle H et al. showed that expression of CXCR3 ligands and CCL5 in chemotherapy tumors did lead to a synergistic increase in T-cell infiltration [36]. It is also been reported that CCL5 released by tumor cells acts its function via paracrine signaling to attract NKs to the tumor bed [37]. Taken together, we assumed that CCL4 and CCL5 may be potent biomarkers and targets for melanoma.

Then, we conducted correlation analysis, and high correlation coefficients were observed among chemokine members (CXCL9, CXCL10, CXCL13, CCL4, CCL5) as expected, suggesting that these chemokines play a synergistic role in the tumorigenesis and progression of SKCM. We also explored the transcription factors for these chemokines, and found that IRF7, RELA, NFKB1, IRF3 and IRF1 may be key transcription factors in the regulation of CCL4, CCL5 and CXCL10. RELA phosphorylation is involved in multiple inflammatory diseases and cancer progression by regulating NF-κB signaling [38]. NFKB1 is an suppressor of inflammation and cancer, which plays an inhibitory role in the occurrence and progression by reducing the abnormal activation of NF-κB signal pathway [39]. IRF family regulates type-I IFN system for anti-tumor immunity through promoting differentiation of B cells, inducing differentiation of naive T cells to effector CD4^+^ or CD8^+^T cells and driving the expression of MHC class I and II [40]. Our results may provide evidence about the complicated correlation among SKCM, chemokines, type-I IFN system and the NF-κB signaling pathway.

In addition, the expressions of CXCL9, CXCL10, CXCL13, CCL4 and CCL5 were positively correlated with six immune cell infiltration (B cells, CD8^+^T cells, CD4^+^T cells, macrophages, neutrophils, dendritic cells) and 28 types of TILs. However, NMU and GAL were not significantly correlated with infiltration of immune cells and TILs. Previous studies suggested that high levels of immune cell infiltration are associated with favorable outcomes [41]. It was similar to our results that increasing levels of B cells, CD8^+^T cells, neutrophils, dendritic cells were related to longer survival time in SKCM patients. A novel study based on thousands of melanoma patients found that the degree of lymphocyte infiltration was an independent prognosticator of disease-free survival (DFS), revealing that a lesser level was associated with a reduced DFS [42]. What’s more, we predicted drug-gene interaction pairs between hub genes and 69 drugs, indicating that our results may reveal immune-related targets for SKCM therapy.

## Conclusion

In summary, our study mainly identified five chemokine members (CCL4, CCL5, CXCL9, CXCL10, CXCL13) and NMU, GAL as significant biomarkers in SCKM tumorigenesis and progression. In addition, their expression levels were closely related to prognosis and immune cell infiltration. This study may provide several potential immune-related targets and help us better understanding the tumorigenesis of SKCM. However, further functional studies are needed to verify the absoluteness of these findings.

## Supplementary information

**Additional file 1:**Summary of up and down-regulated DEGs.

**Additional file 2: Fig. S1.** Correlation of prognostic genes and TILs.

**Additional file 3: Fig. S2.** Survival analysis of six immune cells in SKCM samples.

## Data Availability

The datasets supporting the conclusions of this article are available in the GEO (https://www.ncbi.nlm.nih.gov/geo/) and TCGA repository (https://www.cancer.gov/about-nci/organization/ccg/research/structural-genomics/tcga/studied-cancers).

## Abbreviations

SKCM: Skin cutaneous melanoma
GEO: Gene expression omnibus
DEGs: Differentially expressed genes
PPI: Protein–protein interaction
MCODE: Molecular Complex Detection
GO: Gene Ontology
KEGG: Kyoto Encyclopedia of Genes and Genomes
PM: Primary melanoma
NS: Normal skin
TCGA: The Cancer Genome Atlas
DGIdb: Drug–Gene Interaction database
IHC: Immunohistochemistry
AUC: Area under curve
OS: Overall survival
FDR: False discovery rate
