# Supplementary material for: Identification of immune-related biomarkers associated with tumorigenesis and prognosis in cutaneous melanoma patients
Source: Cancer Cell Int. 2020 May 25;20:195. doi: 10.1186/s12935-020-01271-2 (PMC7249670; doi:10.1186/s12935-020-01271-2)
Supplement: Supplementary file 1 — Additional file 1:Summary of up and down-regulated DEGs. [file 12935_2020_1271_MOESM1_ESM.docx]

| **DEGs Gene names** |
| --- |
| **Up-regulated** AEN KRT17///JUP **CXCL13** RUNX3 ALX1 DUSP4 GMPR SAMSN1 CHST11 CDC42EP3 PPM1H UBD///GABBR1 APBA2 RGS20 DCT OPN3 SNCA MMP1 STMN1 CCL3L3///CCL3L1///CCL3 FCGR2A CCL18 SCG2 CXCL2 ARMC9 PTPRG TNFRSF21 SLC16A4 EN2 HEY1 CDK2 SLAMF7 **CXCL10** TYR CBX3 MARCKSL1 MBP IGSF6 PLP1 FKBP11 SAT1 CAPN3 HRK RNF144A DSC2 MYO10 SNX10 FCGR1B PVRIG LEF1 TRPV2 PLA1A LGALS3BP LZTS1 EDNRB ST3GAL6 SLC45A2 LINC01314 SRGN QPCT IL6 ISG15 IRF4 PMEL BST2 DUSP6 INPP4B SERPINE2 SOX5 MOK CCL8 WIPI1 MAGEA6 PRSS23 TNFRSF10B CDK5R1 C1QB ZNF280B RTP4 CRTAC1 CERS1///GDF1 GPM6B CEP170P1///CEP170 KRT6B CNIH3 CTSZ PEG10 PRAME CDKN2A FOSL1 ARNT2 GAPDHS CSPG4 GDF15 MITF GPR143 MAGEA6///MAGEA3 EVI2A GJB1 PCDH7 ABHD2 QPRT LDHB BAMBI GPR19 BACE2 PHLDA1 SULT1C2 PHACTR1 CTSB SOX10 SPP1 **CCL4** S100B PIK3CD PSEN2 FCMR CHIT1 MIA GYG2 STAT1 BCL2A1 RGS1 MMP11 PLAT GZMB ST3GAL4 SGCD LCP2 **CCL5** ALDH1A3 KCNN2 PTPRM STXBP1 BAALC P2RX7 INSIG1 SOX2 CYBA ADAMDEC1 GREB1 SLC2A3 MIR1908///FADS1 CITED1 PLXNC1 IGHM CTSS WARS MYO5A OSTM1 MC1R TRIB1 MX2 COTL1 GZMA CYTL1 KCNAB2 DOCK10 **CXCL9** MFSD12 MLANA ETV5 LAPTM5 UPP1 RAB27A MLPH TRIB2 |
|  |
| **Down-regulated** CD44 BBOX1 COL4A5 EMX2 POF1B LAMB4 COL7A1 OSR2 KLF4 GJB5 CYP3A5 PAWR MYCL IL18 WIF1 ZNF750 KCNK7 DSC1 SCGB1D2 CA12 GIPC2 EGFR TNXB///TNXA LEPROT///LEPR MEOX2 KLF5 PDZD2 PATJ SEMA3F DCN CYP4B1 HOPX PTGER3 C1orf106 FAT2 FOLR1 PLAGL1 ZBTB16 SLIT3 MRC2 TGM3 SEPT5-GP1BB///SEPT5///GP1BB LINC00302 DSC3 CHP2 ID4 **GAL** EXOSC7///CLEC3B AZGP1 PAMR1 PSORS1C2 COBL CLDN8 ANK3 WNT4 AHNAK2 C1orf116 GATM VAV3 BTG3 KRT23 TACC2 CYP39A1 CXADR DKK2 HOXA10-HOXA9///MIR196B///HOXA9 TRIM29 CDS1 C3orf52 FGFR3 MFAP5 FCGBP NEBL HLF CDHR1 LCE2B BMP2 NPY1R ELL3 IGFBP5 TNFRSF25 EPCAM PPP1R13L ALOXE3 GATA3 PTPRF FBXW7 **NMU** CLDN1 CBLC EVPL EFS LOC101930241///ARHGEF4 AADAC GAN TSPAN8 COL21A1 IRF6 SERPINB7 RAPGEFL1 GJB3 LY6G6C CCL27 TUFT1 FBLN1 MYOF FGFR2 TNNI2 CD207 TPM1 SMPDL3A AGTR1 SCEL SGCG KRT2 MMP28 RORA CLCA4 CLIC3 NFIB IL37 EXPH5 HOOK2 SCGB2A2 DST PAIP2B POU2F3 PLLP FERMT1 PTPN3 SYT17 EPHB6 LRP4 BCL11A PTK6 PTGS1 KLK11 RNF128 TP63 C1orf68 LOC100506718///FLRT2 |

**Supplementary table 1: Identification of the up- and down-regulated overlap DEGs between primary melanoma and normal.**
