# Supplementary figures and images for: Identification of immune-related biomarkers associated with tumorigenesis and prognosis in cutaneous melanoma patients
Source: Cancer Cell Int. 2020 May 25;20:195. doi: 10.1186/s12935-020-01271-2 (PMC7249670; doi:10.1186/s12935-020-01271-2)

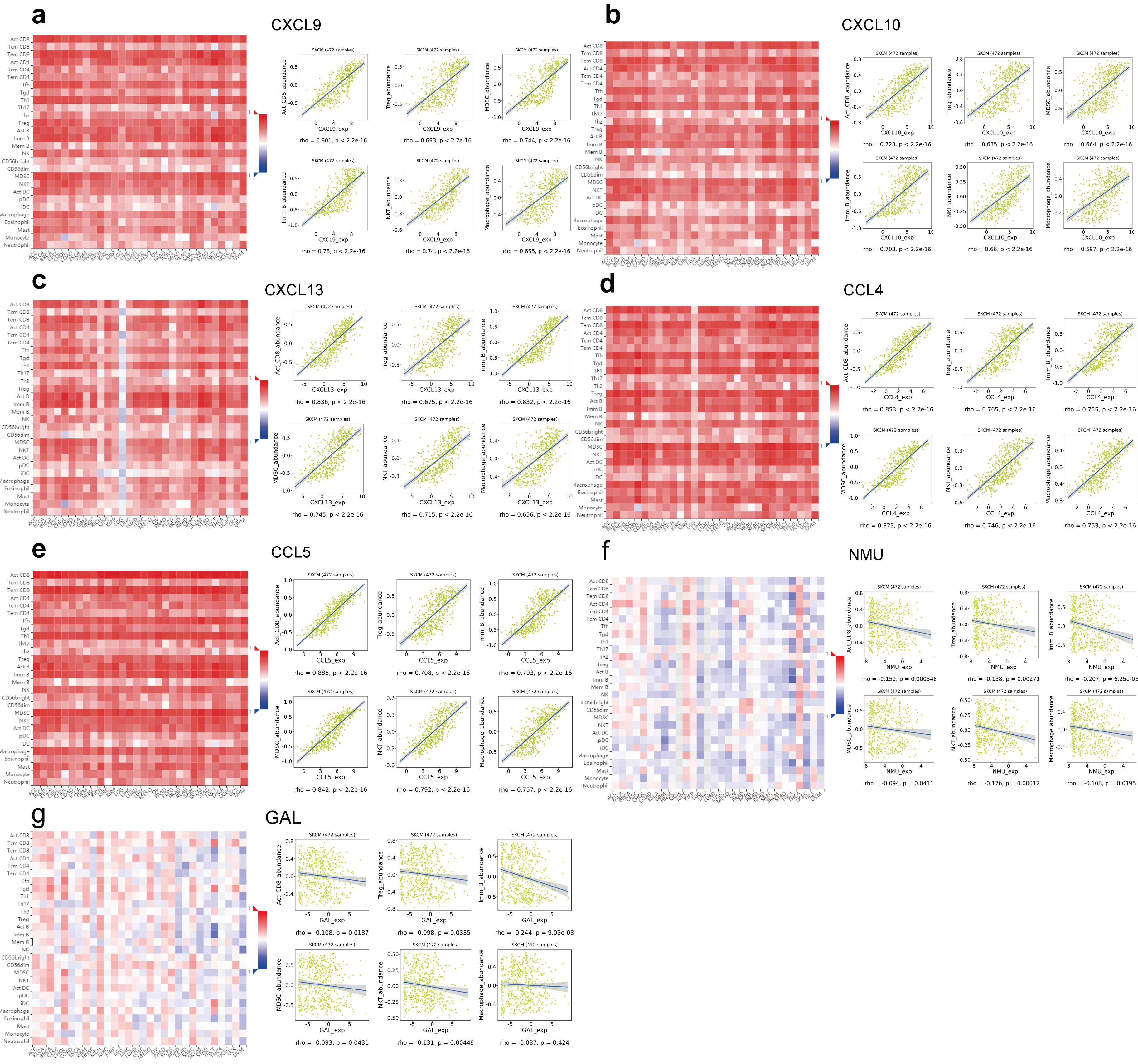

Supplement: Supplementary file 2 — Additional file 2: Fig. S1. Correlation of prognostic genes and TILs. [file 12935_2020_1271_MOESM2_ESM.png]

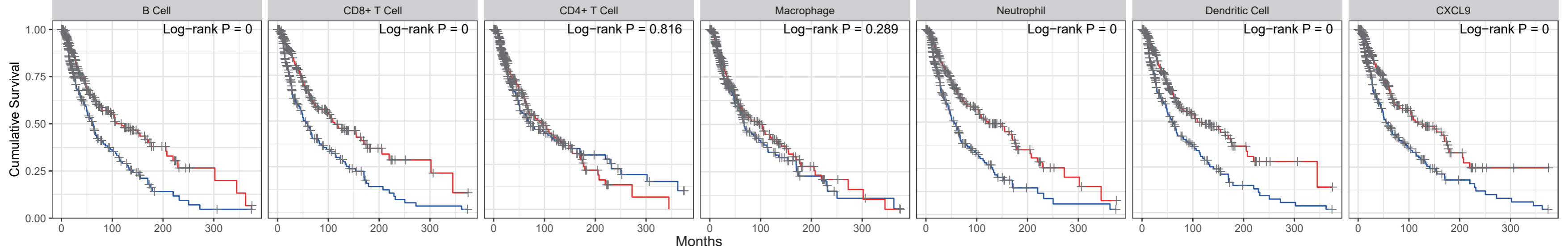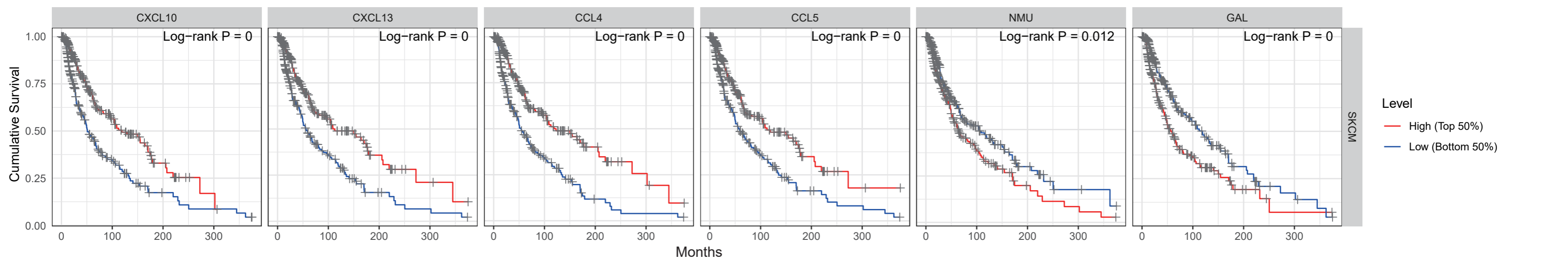

Supplement: Supplementary file 3 — Additional file 3: Fig. S2. Survival analysis of six immune cells in SKCM samples. [file 12935_2020_1271_MOESM3_ESM.pdf]
